# Bayesian Joint Modeling of Multivariate Longitudinal and Survival Data With an Application to Diabetes Study

**DOI:** 10.3389/fdata.2022.812725

**Published:** 2022-04-27

**Authors:** Yangxin Huang, Jiaqing Chen, Lan Xu, Nian-Sheng Tang

**Affiliations:** ^1^College of Public Health, University of South Florida, Tampa, FL, United States; ^2^Department of Statistics, College of Science, Wuhan University of Technology, Wuhan, China; ^3^Department of Statistics, Yunnan University, Kunming, China

**Keywords:** Bayesian inference, longitudinal and survival data, Markov Chain Monte Carlo, multivariate joint models, skew-normal distribution

## Abstract

Joint models of longitudinal and time-to-event data have received a lot of attention in epidemiological and clinical research under a linear mixed-effects model with the normal assumption for a single longitudinal outcome and Cox proportional hazards model. However, those model-based analyses may not provide robust inference when longitudinal measurements exhibit skewness and/or heavy tails. In addition, the data collected are often featured by multivariate longitudinal outcomes which are significantly correlated, and ignoring their correlation may lead to biased estimation. Under the umbrella of Bayesian inference, this article introduces multivariate joint (MVJ) models with a skewed distribution for multiple longitudinal exposures in an attempt to cope with correlated multiple longitudinal outcomes, adjust departures from normality, and tailor linkage in specifying a time-to-event process. We develop a Bayesian joint modeling approach to MVJ models that couples a multivariate linear mixed-effects (MLME) model with the skew-normal (SN) distribution and a Cox proportional hazards model. Our proposed models and method are evaluated by simulation studies and are applied to a real example from a diabetes study.

## 1. Introduction

In epidemiologic and clinical studies, a lot of attention is focused on developing the specific patterns of the longitudinal measurements and the associations between those patterns and the time to a certain event, such as diagnosis of disease, time to transplantation, or death. Those studies have been in a highly active research area (Henderson et al., 2000; Brown and Ibrahim, 2003; Tsiatis and Davidian, 2004; Rizopoulos, 2011, 2012). For example, in diabetes studies, repeated measures of continuous exposures such as the children's growth (height and weight) and time to type 1 diabetes (T1D) are collected.

**Figure 1:**
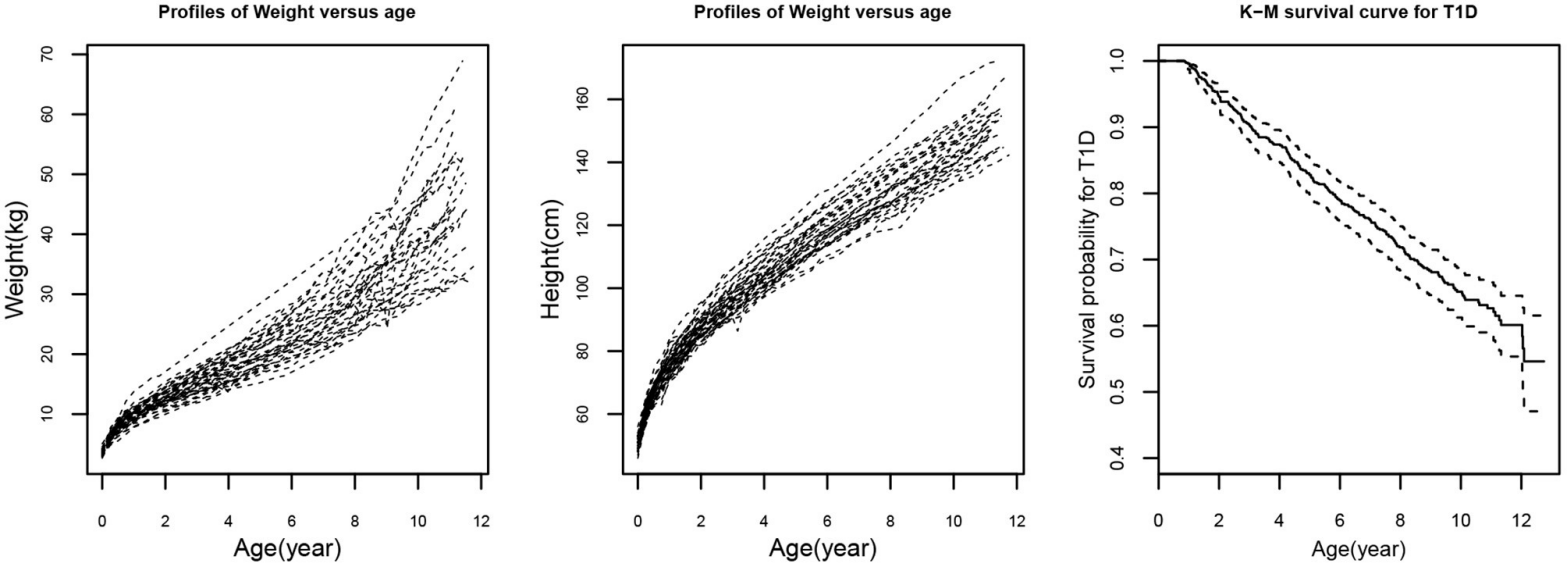
Randomly selected 50 trajectories of weight (left panel) and height (middle panel) from a diabetes study. Kaplan-Meier (K-M) survival plot (right panel) for type 1 diabetes (T1D).

During the last two decades, the research on joint modeling of longitudinal and time-to-event data has been received rapid and considerable development. In literature, various joint models and associated statistical methods have been introduced to analyze such longitudinal and survival data. However, the following issues may stand out. (i) Most joint models focus on a single longitudinal variable associated with a time-to-event outcome (Henderson et al., 2000; Brown and Ibrahim, 2003; Tsiatis and Davidian, 2004; Rizopoulos, 2011, 2012). However, in practice, many studies often collect multiple longitudinal outcomes (Lin et al., 2002; Brown et al., 2005; Chi and Ibrahim, 2006; Fieuws and Verbeke, 2006; Albert and Shih, 2010; Rizopoulos and Ghosh, 2011; Kim and Albert, 2016; Chen and Wang, 2017; Tang et al., 2017a,b; Proudfoot et al., 2018; Chen et al., 2021) which may be significantly correlated. For example, the weight and height repeated measures presented in Figure 1 (left and middle panels) show significant correlation and it may lead to biased estimation if their correlation is ignored. In addition, time-to-event such as the time to T1D depicted in Figure 1 (right panel) may be dependent on the longitudinal weight and height measures. (ii) In traditional linear mixed-effects models, within subject measurement errors are often under a normality assumption due to the mathematical tractability and computational convenience. However, the normality assumption may not be realistic. Alternatively, the skew-elliptical (SE) distributions including skew-normal (SN) distribution (Sahu et al., 2003) should be more appropriate to model the skewed data (Azzalini and Capitanio, 2003; Sahu et al., 2003; Arellano-Valle and Genton, 2005; Huang and Dagne, 2011). Although a few studies investigated multivariate joint (MVJ) models (Chi and Ibrahim, 2006; Albert and Shih, 2010; Rizopoulos and Ghosh, 2011; Kim and Albert, 2016; Chen and Wang, 2017; Tang et al., 2017a,b; Chen et al., 2021), they have not considered non-normal features of longitudinal data.

In statistical literature, longitudinal data analysis has focused on developing models to capture only specific aspects of the motivating studies. Inferential procedures may be complex dramatically if one considers multiple longitudinal outcomes data with correlation and skewed distributions in conjunction with an event time in MVJ models. Our Bayesian approach enables the fitting of such models efficiently and the convergence problem can be solved.

The rest of the article is organized as follows. In Section 2, we introduce MVJ models with the SN distribution and discuss the associated Bayesian inferential method. In Section 3, we present the data set from a diabetes study that motivated this research, apply the specific MVJ model to the data, and report the results. Section 4 conducts limited simulation studies to evaluate the performance of the proposed models and method. Finally, a general discussion and conclusion are presented in Section 5.

## 2. Joint Models and Associated Bayesian Approach

This section presents the MVJ model and related Bayesian modeling method in full generality for multiple longitudinal data with non-normality and correlation and survival endpoint with censoring to illustrate that our modeling method can be applied in various applications. To relax the normality assumption, the multivariate longitudinal model with SN distribution is assumed. Let *y*_*ijk*_ denote an observation of the *kth* longitudinal variable (*k* = 1, 2, …, *K*) for the *ith* subject (*i* = 1, 2, …, *n*) at the *jth* visit time *t*_*ijk*_ (*j* = 1, 2, …, *n*_*i*_). Let $Y_{i}={\left(Y_{i1}^{T},\ldots ,Y_{iK}^{T}\right)}^{T}$ be the *K*-variate vector of continuous longitudinal responses, where $Y_{ik}={\left(y_{i1k},\ldots ,y_{in_{i}k}\right)}^{T}$. Similarly, we can define ***X***_*i*_ and ***Z***_*i*_. Let the vector of population parameters $\beta ={\left(\beta_{1}^{T},\ldots ,\beta_{K}^{T}\right)}^{T}$ with $\beta_{k}^{T}={\left(\beta_{0k},\beta_{1k},\beta_{2k},\ldots ,\beta_{pk}\right)}^{T}$ associated with the *k*^*th*^ longitudinal variable. The vector of subject-specific parameters by $b_{i}={\left(b_{i1}^{T},\ldots ,b_{iK}^{T}\right)}^{T}$ with $b_{ik}^{T}={\left(b_{i0k},b_{i1k},b_{i2k},\ldots ,b_{iqk}\right)}^{T}$. We denote $T_{i}^{*}$ as the ‘event' time, *C*_*i*_ as the censoring time and $T_{i}=min\left(T_{i}^{*},C_{i}\right)$ as the observed time for subject *i*. Let ρ_*ij*_ denote the indicator for an event, i.e., ρ_*ij*_ = 0 when censoring occurs and ρ_*ij*_ = 1 $\left(T_{i}^{*}\leq C_{i}\right)$ when the event is observed. ***x***_*i*_ is a covariate vector that may be associated with an event time.

### 2.1. Multivariate Linear Mixed Effects Models With SN Distribution

We consider a general multivariate linear mixed-effects (MLME) model with SN distribution as follows.


(1) $$\begin{array}{l} \begin{array}{c} Y_{ik}=X_{ik}\beta_{k}+Z_{ik}b_{ik}+\epsilon_{ik},\\ b_{i}={\left(b_{i1}^{T},\ldots ,b_{iK}^{T}\right)}^{T}\overset{\text{iid}}{\sim }N_{Kq}\left(0,\Sigma_{b}\right),\\ \epsilon_{i}={\left(\epsilon_{i1}^{T},\ldots ,\epsilon_{iK}^{T}\right)}^{T}\overset{\text{iid}}{\sim }\\ SN_{Kn_{i}}\left(-\sqrt{2/\pi }\left[\Delta_{K}⊗1_{n_{i}}\right],\Sigma_{K}⊗I_{n_{i}},\Delta_{K}⊗I_{n_{i}}\right), \end{array} \end{array}$$

where the random error vector **ϵ**_*i*_ follows a multivariate SN distribution with unknown variance-covariance matrix $\Sigma_{K}={\left(\sigma_{kk^{\prime }}^{2}\right)}_{K\times K}$ (*k, k*′ = 1, 2, …, *K*, unknown skewness parameter matrix **Δ**_*K*_ = diag(δ_1_, …, δ_*K*_), the skewness parameter vector $\delta_{K}={\left(\delta_{1},\ldots ,\delta_{K}\right)}^{T}$, and $1_{n_{i}}={\left(1,\ldots ,1\right)}^{T}$. Note that $-\sqrt{2/\pi }\left[\Delta_{K}⊗1_{n_{i}}\right]$ is considered here in order to make the SN distribution with mean zero; refer to (Sahu et al., 2003; Huang and Dagne, 2011; Xu, 2021) for a detailed discussion of SN distribution. The vector of random-effects ***b***_*i*_ follows *N*_*Kq*_(**0**, **Σ**_*b*_) with **Σ**_*b*_ being a covariance matrix. In the application below, we are interested in the height and weight longitudinal data. Let **Δ**_2_ = diag(δ_1_, δ_2_), and δ_1_ and δ_2_ quantify skewness of height and weight, respectively, which is the case represented in this article.

### 2.2. Cox Proportional Hazard Model

In survival analysis, the semiparametric Cox hazard model (Cox, 1972; Xu, 2021) has been commonly adopted to explore the association between survival time and one or more covariates in medical research. To account for the association between the multiple longitudinal exposures, we assume that the distribution of *T*_*i*_, the time to diagnosis of T1D for subject *i*, depends on the random-effects of individual-specific longitudinal processes ***b***_*ik*_, and other covariates ***x***_*i*_, respectively. The survival model for *T*_*i*_ here is linked to the multivariate longitudinal model (6) through the random-effects ***b***_*i*_. In addition, assuming that the covariates ***x***_*i*_ are associated with the event time. In particular, the conditional hazard rate of *T*_*i*_ at time *t*_*i*_ for the survival component is given by Xu (2021).


(2) $$\begin{array}{l} \begin{array}{c} \lambda \left(t_{i}|b_{i},x_{i}\right)=\lambda_{0}\left(t_{i}\right)\exp\left(\Upsilon^{T}b_{i}+\alpha^{T}x_{i}\right)=\lambda_{0}\left(t_{i}\right)\exp\left(\gamma^{T}d_{i}\right), \end{array} \end{array}$$

where λ_0_(*t*_*i*_) is the baseline hazard function, $d_{i}={\left(b_{i}^{T},x_{i}^{T}\right)}^{T}$, **γ** = (Υ^*T*^, **α**^*T*^)*T*, Υ and **α** are unknown parameters linked with the covariates ***x***_*i*_ and random-effects ***b***_*i*_ to the conditional hazard rate, respectively. The association parameter vector Υ linking the random-effects ***b***_*i*_ measures the association between the two sub-models. An alternative method can be used to approximate the Cox proportional hazards model (2) through the counting process (Clayton, 1991) which is adopted for our joint modeling and can obviously reduce computational burdens; a detailed discussion of the alternative method can be found in Huang (2016), Huang and Chen (2016), and Zhang and Huang (2021).

### 2.3. Simultaneous Bayesian Inferential Approach

Generally, different approaches are applied to link the longitudinal and survival submodels. The first approach is under the framework of the likelihood inferential methods, such as the Expectation-Maximization (EM) algorithm and Monte Carlo Expectation-Maximization (MCEM) algorithm (Rizopoulos et al., 2010; Farcomeni and Viviani, 2015; Xu, 2021). A simultaneous inferential method through a joint likelihood may be favorable, but the computational burden for proposed MVJ modelings can be very intensive, even sometimes infeasible, and may cause problems of algorithm convergence (Brown and Ibrahim, 2003; Wu et al., 2010). The second approach is Bayesian inference, Bayesian joint modeling method shows the advantage. Thus, the parameters can be estimated simultaneously for the MVJ models implemented by Markov Chain Monte Carlo (MCMC) techniques for the skew-normal MVJ model. The simultaneous statistical inference on all unknown model parameters will capture the underlying association between the longitudinal exposures and the event time data.

First, ***b***_*ik*_ and **ϵ**_*ik*_ are assumed mutually independent of each other. To specify the Model (1) for MCMC computational techniques, by introducing the *n*_*i*_-dimensional random vector ***w***_*i*_ based on the stochastic representation of SN distribution detailed in the publication (Huang and Dagne, 2011), we can hierarchically formulate the MVJ model, which consists of the MLME Model (1) and Cox proportional hazard Model (2) as follows.


(3) $$\begin{array}{l} \begin{array}{c} Y_{i}|b_{i},w_{i}\sim N\left(X_{i}\beta +Z_{i}b_{i}+\Delta_{K}⊗\left[w_{i}-\sqrt{2/\pi }1_{n_{i}}\right],\Sigma_{K}⊗I_{n_{i}}\right),\\ w_{i}\sim N_{i}\left(0,I_{n_{i}}\right)I\left(w_{i}>0\right),b_{i}\sim N_{Kq}\left(0,\Sigma_{b}\right),\\ T_{i}\sim F\left(t_{i}|d_{i},\lambda_{0}\right)=\int f\left(\rho_{i}|b_{i},x_{i}\right), \end{array} \end{array}$$

Then, under the Bayesian framework, we need to specify **θ** = (**β**, **γ**, **Σ**_*b*_, **Σ**_*K*_, **δ**_*K*_) as all unknown population parameters in the joint Model (1) and (2), where $\delta_{K}={\left(\delta_{1},\ldots ,\delta_{K}\right)}^{T}$ is the vector of skewness parameters. We assign weakly informative priors to ensure the property of posteriors. Thus, the prior distributions for all of the unknown parameters are specified as follows.


(4) $$\begin{array}{l} \begin{array}{c} \beta \sim N\left(\beta_{0},\Omega_{1}\right),\gamma \sim N\left(\gamma_{0},\Omega_{2}\right),\Sigma_{b}\sim \text{IW}\left(\Omega_{3},\omega_{1}\right),\\ \Sigma_{K}\sim \text{IW}\left(\Omega_{4},\omega_{2}\right),\delta_{K}\sim N\left(0,\Omega_{5}\right), \end{array} \end{array}$$

where the mutually independent Normal (*N*) and Inverse Wishart (*IW*) prior distributions are chosen to facilitate computations. The super-parameter matrices **Ω**_1_, **Ω**_2_, **Ω**_3_, **Ω**_4_ and **Ω**_5_ can be assumed to be diagonal for convenient implementation.

Subsequently, let *f*(·), *f*(·|·), *F*(·|·), and π(·) denote a density function, a conditional density function, a cumulative density function (c.d.f), and a prior density function, respectively. As the elements of **θ** = {**β**, **γ**, **Σ**_*b*_, **Σ**_*K*_, **δ**_*K*_} are assumed to be independent of each other, we have π(**θ**) = π(**β**)π(**γ**)π(**Σ**_*b*_)π(**Σ**_*K*_)π(**δ**_*K*_). After we specify the MVJ model for the observed data and the prior distributions for the unknown parameters, we can make the Bayesian inference for the parameters based on their posterior distributions. Thus, the joint posterior density of **θ** based on the observed data $D=\left\{Y_{i},b_{i},\rho_{i}\right\}$ can be represented by


(5) $$\begin{array}{l} f(\theta |D)\propto \{\prod_{i=1}^{n}\int f(Y_{i}|b_{i},w_{i})f(b_{i})\\ f(w_{i}|w_{i}>0)f(\rho_{i}|b_{i},x_{i})db_{i}\}\pi (\theta ). \end{array}$$

In general, the integrals in (5) do not have a closed form and are high dimensional. Since the approximated analysis of the integrals may not be sufficiently accurate, it is prohibitive to directly compute the posterior distribution of **θ** from the observed data. Alternatively, the MCMC technique can be used for sampling random-effects ***b***_*i*_ and population parameters **θ** from conditional posterior distributions based on (5), by employing the Gibbs sampler with the Metropolis-Hastings (M-H) algorithm together. We repeat this process in iterations of the MCMC algorithm until convergence is achieved by adopting the publicly available WinBUGS package (Lunn et al., 2000). An advantage of the WinBUGS package is that it does not require explicitly deriving the full conditional posterior distributions for unknown parameters. Although their derivations are straightforward based on the joint posterior (5), they are not presented here to save space due to some cumbersome algebra.

## 3. Application

### 3.1. Motivating Data Set

The motivated data set is from a diabetes study, which is a prospective multinational (U.S., Finland, Sweden, and Germany) cohort to investigate the environmental determinants of T1D (TEDDY Study Group, 2007; Larsson et al., 2008; Chen et al., 2021). This study recruited both the first degree relative (FDR) children and the general population to be screened for genetic predisposition for T1D-related Human Leukocyte Antigen-antigen D and isotype R (HLA-DR) genotypes at the time of birth when they are eligible. The details on the characteristics of families of the diabetes cohort have been reported (Lernmark et al., 2011; Baxter et al., 2012). The participants enrolled in this diabetes study are followed prospectively from birth to 15 years old, with study visits beginning at 3 months of age, then every 3 months until 4 years of age, and every 6 months thereafter depending on the development of T1D. The details of screening and follow-up have been published previously (Kiviniemi et al., 2007; Hagopian et al., 2011). The collected data at each visit time include repeated height and weight measurements, time-to-event outcomes such as the first sign of islet autoimmunity (IA) and clinical diagnosis of T1D, biological data, dietary records, demographic and health histories for the children, and psychological measurements (TEDDY Study Group, 2007). T1D is a common pediatric chronic disease and is preceded by a preclinical period of IA in the presence of islet autoantibodies.

In this real data application here, we consider the dataset of 732 children from all subjects who have developed IA which is the preclinical sign for potentially clinical diagnosis of T1D and have repeated weight and height values from birth to age at diagnosis of T1D or most recent visit. The confirmed T1D is defined as confirmed positive antibodies to insulin, insulinoma antigen 2, or glutamic acid decarboxylase, which are analyzed by a radiobinding assay at least 2 consecutive visit times (Larsson et al., 2008; Elding Larsson et al., 2014; Chen et al., 2021). For children with an event that occurred, only repeated measurements up to the date of diagnosis of T1D are included in the analysis, while for subjects with censoring, repeated measurements up to the age of 15 are used. A child's growth trajectory in early life shows a quadratic pattern approximately as displayed in Figure 1 (left and middle panels). The pattern of a subject may be an important clinical implication because of its association with the T1D risk. Figure 1 (right panel) shows the Kaplan-Meier (K-M) survival curve for T1D as the event. Among the 732 subjects, 246 (33.61%) children are progressed to T1D. The main risk factors used in the analysis include gender (women vs. men), country of residence (Finland, Germany, Sweden as compared to the United States FDR status (yes or no), and HLA genotype (HLA-DR3/4 genotype compared with others) which are the most important genetic and environmental factors in this diabetes study (Larsson et al., 2008; Chen et al., 2021).

### 3.2. Model Implementation

We illustrate our models and method for the part of longitudinal data described in Section 3.1. We used an SN MLME model with random intercept, random slope, and quadric of age and gender for the longitudinal submodel and adjusted for random intercept and random slope, gender, HLA genotype, and FDR status at baseline in the survival submodel. We consider the following specific bivariate linear mixed-effects models for height and weight:


(6) $$\begin{array}{l} \begin{array}{c} y_{ijk}=\left(\beta_{0k}+b_{i0k}\right)+\left(\beta_{1k}+b_{i1k}\right){\text{Age}}_{ij}\\ \;+\beta_{2k}{\text{Age}}_{ij}^{2}+\beta_{3k}{\text{Gender}}_{i}+e_{ijk},\\ \;\text{for }k=1,2, \end{array} \end{array}$$

Specifically, where *k* = 1 and 2 correspond to the respective height and weight responses. *y*_*ij*1_ and *y*_*ij*2_ are the respective standardized height and weight observations for the *ith* subject at time *t*_*ij*_; the random-effects *b*_*i*0*k*_ and *b*_*i*1*k*_ represent a subject-specific random intercept and a subject-specific random slope, respectively. In this model, the mean baseline value (intercept), mean change rate (slope), and quadratic of age are assumed to be different between men and women.

The survival analysis of the joint model is explained in Section 2.3. The Cox proportional hazard model applied in our study is specified as follows.


(7) $$\begin{array}{l} \lambda (t_{i}|b_{i},x_{i})\;=\;\lambda_{0}(t_{i})exp(\upsilon_{1}b_{0i1}\;+\;\upsilon_{2}b_{1i1}\;+\;\upsilon_{3}b_{0i2}\\ \;+\;\upsilon_{4}b_{1i2}\;+\;\alpha_{1}{\text{Finland}}_{i}\;\\ \;+\;\alpha_{2}{\text{Germany}}_{i}\;+\;\alpha_{3}{\text{Sweden}}_{i}\;+\;\alpha_{4}{\text{Gender}}_{i}\\ \;+\;\alpha_{5}{\text{HLA}}_{i}\;+\;\alpha_{6}{\text{FDR}}_{i}). \end{array}$$

where Υ = (υ_1_, υ_2_, υ_3_, υ_4_) is the parameters corresponding to the random-effects ***b***_*i*_ = (*b*_*i*01_, *b*_*i*11_, *b*_*i*02_, *b*_*i*12_) and other risk factors are included in the survival model. Additionally, **α** = (α_1_, …, α_6_) is corresponding to the risk factors including country of residence (Finland, Germany, and Sweden), gender (female = 1), HLA genotype, and FDR status.

In the diabetes study data, height and weight variables exhibit skewness and outliers. Based on the nature of the diabetes study data, the two statistical models with different distributions are implemented to compare their performance as follows.

- **Model N:** MVJ model with the normal distribution for random errors.
- **Model SN:** MVJ model with the SN distribution for the random errors.

Because a normal distribution is a special case of an SN distribution when the skewness parameter becomes zero, we explore how the MVJ model with an SN distribution contributes to modeling results and parameter estimates in comparison with that with a symmetric normal distribution.

In order to perform the Bayesian inferential method, we need to specify the values of the hyper-parameters in the prior distributions (4). Due to the absence of historical data, we apply weakly informative prior distributions for the parameters in MVJ models. In particular, (i) fixed-effects are taken to be independent normal distribution *N*(0, 0.01) for each element of the population parameter vectors **β**, **υ** and **α**; (ii) the priors for the variance covariance matrices **Σ**_*K*_ and **Σ**_*b*_ follow IW distributions *IW*(diag(0.01, 0.01), 2) and *IW*(diag(0.01, 0.01, 0.01, 0.01), 4); (iii) for each of the skewness parameters δ_1_ and δ_2_, which represent the skewness of height and weight, respectively, independent normal distributions *N*(0, 0.01) is chosen.

**Figure 2:**
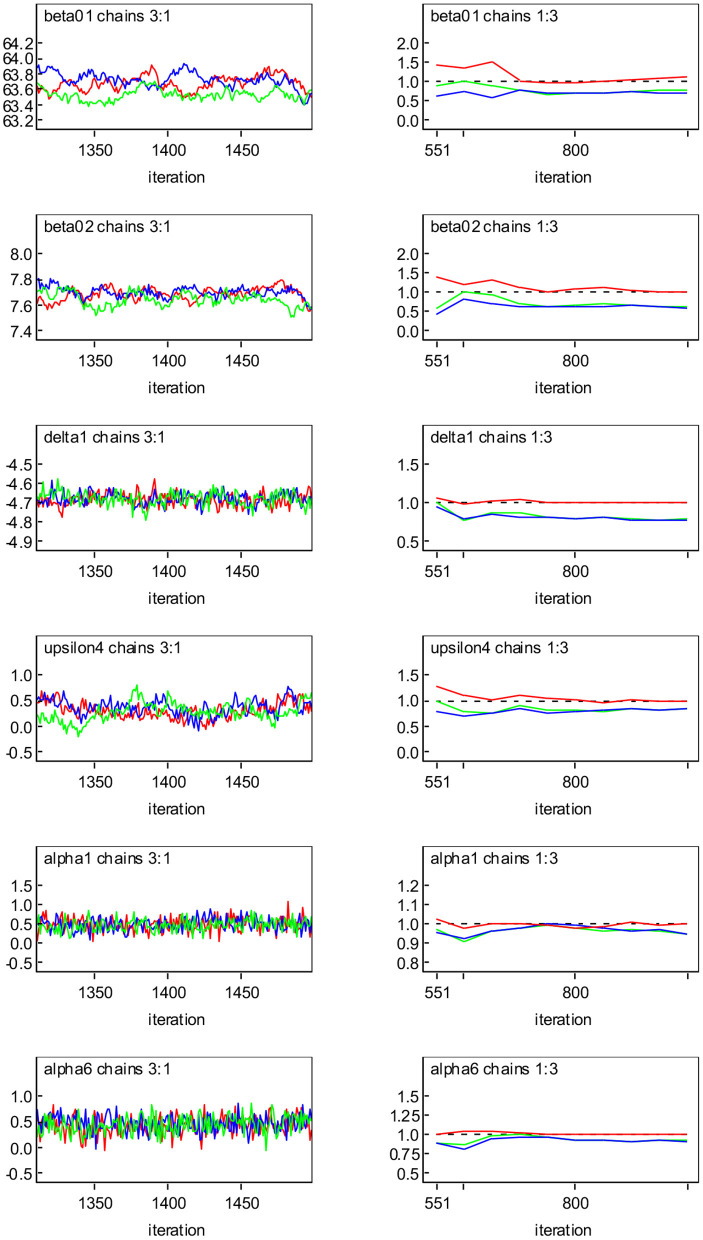
Convergence diagnostics with three Markov chains for representative parameters based on Model SN: trace plots (left panel); Gelman-Rubin (GR) diagnostic plots (right panel), where the middle and bottom curves below the dashed horizontal line (indicated by the value one) represent the pooled posterior variance ($\hat{V}$) and average within-sample variance (Ŵ), respectively, and the top curve above the dashed horizontal line represents their ratio ($\hat{R}$).

The MCMC sampler is implemented and the program codes are provided in Appendix. When the MCMC algorithm is applied to the diabetes study data, the convergence of the generated samples is assessed using standard tools such as Gelman-Rubin (GR) diagnostics (Gelman and Rubin, 1992) and trace plots. Figure 2 shows the dynamic version of GR diagnostic plots and the trace plots based on Model SN for the representative parameters β_01_, β_02_, δ_1_, υ_4_, α_1_ and α_6_. We can see from trace plots (left panel) that the lines of three different chains mix or cross in the trace, indicating that the algorithmic convergence is achieved. For the plots of GR diagnostics (right panel) where the three curves are given. The top curve indicates the ratio ($\hat{R}$) of the middle curve and the bottom curve below the dashed horizontal line (indicated by the value 1) which represent, respectively the pooled posterior variance ($\hat{V}$) and average within-sample variance (Ŵ). It can be seen that $\hat{R}$ is generally higher than one at the initial stage of the algorithms, but it tends to 1 eventually, and $\hat{V}$ and Ŵ tend to stabilize as the number of iterations increases, suggesting that the MCMC algorithm has approached convergence.

When these criteria indicate the algorithm convergence of chains, we propose that, after an initial number of 10,000 burn-in iterations of three chains of length 30,000, every 20th MCMC sample was retained from the next 10,000 for each chain. Thus, we obtain a total of 3,000 samples for targeted posterior distributions of the unknown parameters for statistical inference. Even though this is a high-dimensional computation working load, the MCMC algorithm has no problem regarding the convergence of a solution for the inverse of matrices and parameter estimates in this application.

### 3.3. Data Analysis Results

**Figure 3:**
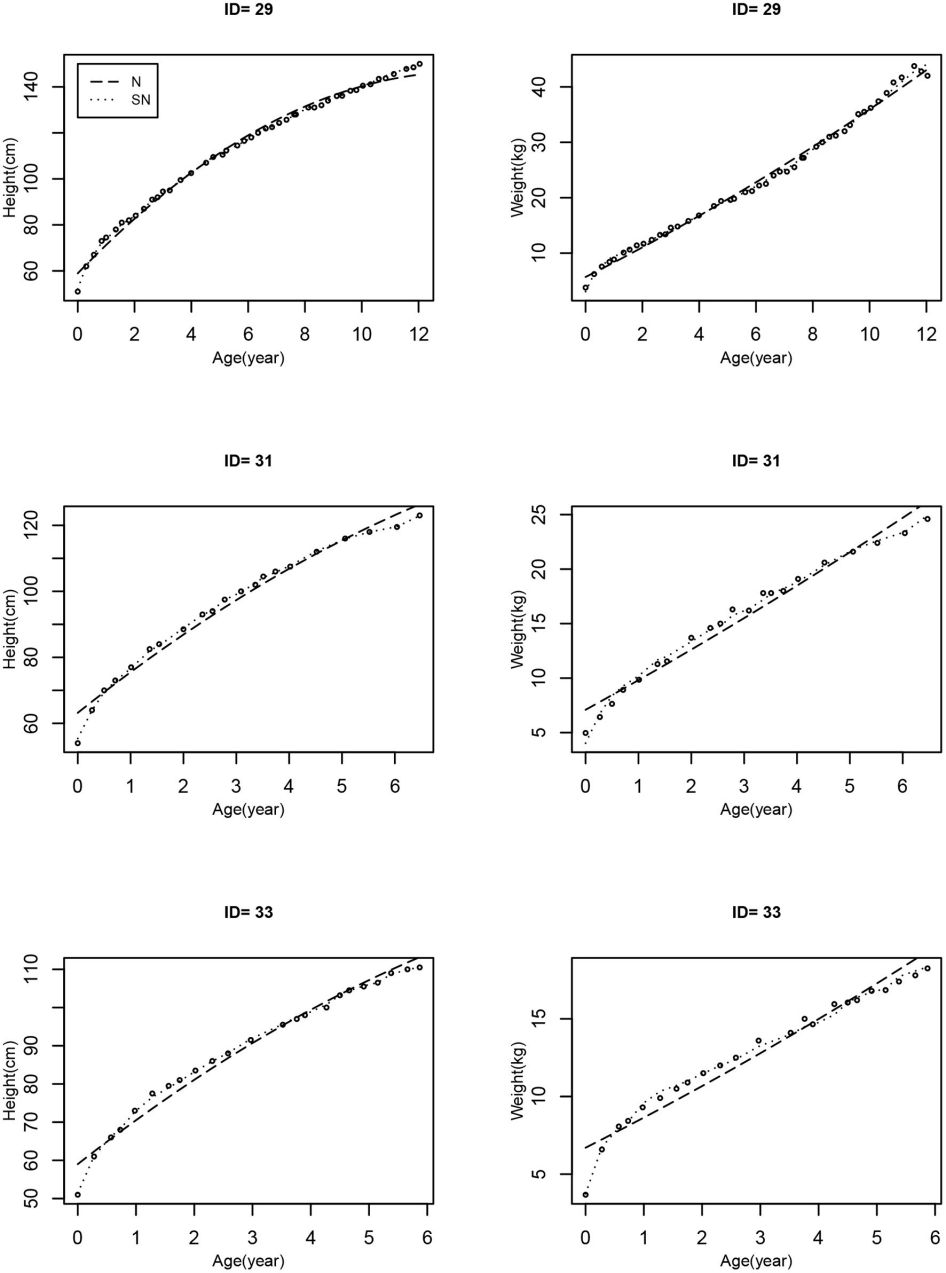
The individual estimates of height and weight trajectories for 3 randomly selected patients based on the two models (Model N: dashed line; Model SN: dotted line). The observed values are indicated by circles.

Bayesian joint modeling approach based on MLME models is used to fit height and weight, as well as time-to-event data jointly. From the model fitting results, we have seen that, in general, the longitudinal sub-model provides a reasonably good fit to the observed data for most participants in the study; in particular, Figure 3 shows the three randomly selected individual fitting curves of the height and weight trajectories estimated by the MVJ modeling approach based on Models N and SN. We can see that the estimated individual curves for Model SN, where the model error follows the SN distribution, fit the observed data more closely than those for Model N where the model error is normally distributed. The following findings are obtained from MVJ modeling.

**Figure 4:**
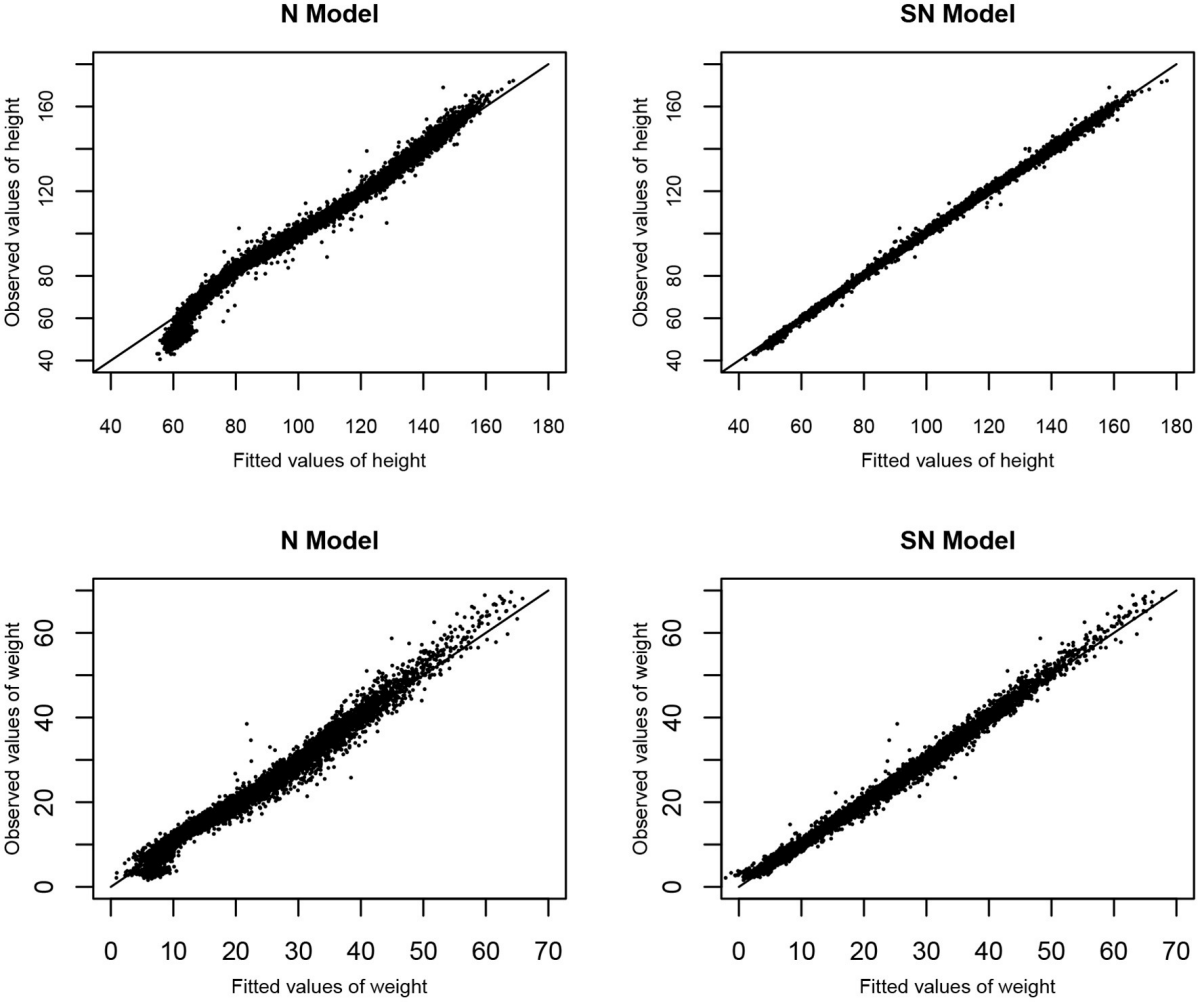
The goodness of fit: Observed values verse fitted values of height and weight based on Models N and SN.

To access the goodness-of-fit of the two models based on the MVJ modeling approach, the diagnostic plots of observed values vs. fitted values of height and weight based on Models N and SN are represented in Figure 4. It is shown from Figure 4 that Model SN provides a much better fit to the observed values of height and weight, as compared to Model N.

Table 1 presents the population posterior mean (PM), the corresponding standard deviation (SD), and 95% equal-tail credible interval (CI) for the fixed-effects parameters and Cox proportional hazard model parameters based on Models SN and N. The following are our findings for the results of estimated parameters. First, in the multivariate longitudinal model (6), the results present that these estimates are different from zero since 95% of CIs do not contain zero. In particular, for the parameters that are of interest, the estimates of β_11_ and β_12_ for the growth rate of height and weight, respectively, for Model SN, are slightly smaller than their counterparts for Model N, while the baseline estimates of β_01_ and β_02_ from Model SN are slightly larger than those from Model N. Second, the estimates of the within-subject variances $\sigma_{11}^{2}$, $\sigma_{22}^{2}$, and covariance $\sigma_{12}^{2}$ for Model SN are smaller than their counterparts for Model N. This is expected because of high variability, heaviness of the tails, and skewness are pertinent to certain criteria. The estimates of the skewness parameters δ_1_ = −4.68 and δ_2_ = −1.82 are significantly negative for Model SN. The results provide evidence of obvious left-skewness exists in our data. Consequently, Model SN containing the skewness parameters is recommended. Third, there is an interesting finding in the Cox proportional hazard model (7) for the time-to-event process. Model SN indicates that the estimated two association parameters of the height [−0.27 with 95% CI (−0.33−0.21) and 0.95 with 95% CI (0.69, 1.22)], and one association parameter of weight [0.42 with 95% CI 0.42(0.25, 0.62)] are significantly associated with the risk of T1D. In the comparison of Model SN and Model N, there is not too much difference in the parameter estimates of the Cox proportional hazard model. The estimated results also show that it is not directly associated with the covariates of Sweden and gender because of the insignificant estimates of α_3_ and α_4_.

**Table 1:** Summary of the estimated posterior mean (PM) and standard deviation (SD) of the population (fixed-effects) parameters, and the corresponding 95% equal-tail credible interval (CI) as well as deviance information criterion (DIC) values.

**Parameter**	**Model SN**			**Model N**		
	**PM**	**SD**	**95% CI**	**PM**	**SD**	**95% CI**
**Parameter estimates in longitudinal sub-model**						
β_01_	63.63	0.13	(63.38, 63.88)	61.89	0.12	(61.66, 62.15)
β_11_	11.69	0.05	(11.6, 11.8)	12.74	0.04	(12.66, 12.81)
β_21_	–0.37	0.01	(–0.38, -0.36)	-0.467	0.01	(–0.473, –0.461)
β_31_	–1.55	0.21	(–1.96, –1.15)	–1.52	0.17	(–1.87, –1.18)
β_02_	7.67	0.07	(7.53, 7.79)	7.01	0.06	(6.89, 7.13)
β_12_	2.13	0.03	(2.07, 2.19)	2.53	0.06	(2.47, 2.59)
β_22_	0.079	0.01	(0.077, 0.082)	0.044	0.01	(0.041, 0.046)
β_32_	–0.58	0.09	(–0.76, –0.40)	–0.57	0.08	(–0.73, -0.41)
δ_1_	–4.68	0.03	(–4.75, –4.61)	–	–	–
δ_2_	–1.82	0.03	(–1.86, –1.77)	–	–	–
$\sigma_{11}^{2}$	1.21	0.05	(1.12, 1.33)	9.99	0.11	(9.78, 10.21)
$\sigma_{12}^{2}$	–0.44	0.02	(–0.48, –0.39)	2.96	0.04	(2.87, 3.04)
$\sigma_{22}^{2}$	0.74	0.02	(0.74, 0.79)	2.06	0.02	(2.01, 2.10)
**Parameter estimates in survival sub-model**						
υ_1_	–0.27	0.03	(–0.33, –0.21)	–0.35	0.04	(–0.43, –0.27)
υ_2_	0.95	0.13	(0.69, 1.22)	1.26	0.18	(0.94, 1.67)
υ_3_	0.42	0.09	(0.25, 0.62)	0.43	0.09	(0.26, 0.63)
υ_4_	0.32	0.17	(–0.027, 0.65)	0.29	0.18	(–0.07, 0.65)
α_1_	0.51	0.17	(0.18, 0.84)	0.41	0.18	(0.04, 0.41)
α_2_	0.58	0.26	(0.037, 1.08)	0.51	0.18	(0.01, 0.52)
α_3_	–0.15	0.18	(–0.51, 0.19)	–0.34	0.18	(-0.70, 0.03)
α_4_	–0.054	0.14	(–0.33, 0.21)	–0.03	0.14	(-0.31, 0.25)
α_5_	0.34	0.14	(0.061, 0.62)	0.38	0.14	(0.09, 0.66)
α_6_	0.43	0.17	(0.076, 0.76)	0.45	0.18	(0.10, 0.79)
DIC	122,274			160,068		

In order to select the best model which fits the data adequately, we adopt a Bayesian selection criterion, known as the deviance information criterion (DIC) (Spiegelhalter et al., 2002). As we know, DIC is not intended to identify the “correct" model but is only used to find the one that fits the data best. the DIC values obtained are also summarized in Table 1 in order to compare models under different settings. It is seen that the DIC value in Model SN is smaller than its counterpart in Models N, suggesting that Model SN produces a better fit than Model N in terms of DIC value. Thus, the further results based on Model SN are reported in detail below.

The estimated results based on Model SN in Table 1 suggest the skewness parameters in height (−4.68) and weight (−1.82) are estimated to be significantly negative. This suggests the skewness with the heavy left tail of height and fairly left tail of weight. Thus, it may suggest that accounting for a multivariate linear mixed-effects joint modeling with the SN distribution offers a better fit to the data which exhibit skewness and, in turn, provides more reliable parameter estimates. The estimated results of fixed-effects presented in Table 1 based on Model SN indicate that the growth rate of height and weight with quadratic terms of age and gender may be approximated by $\hat{y_{1}}=63.63+11.69\times \text{Age}-0.37\times {\text{Age}}^{2}-1.55\times \text{Gender}$ and $\hat{y_{2}}=7.67+2.13\times \text{Age}+0.079\times {\text{Age}}^{2}-0.58\times \text{Gender}$, respectively. The quadratic of age and gender are all significant for the longitudinal sub-model of the MVJ modeling. Finally, based on the survival sub-model, the results show that the hazard ratio of the estimated association parameter υ_2_ is exp(υ_2_) = 2.59 with 95% CI being (1.99, 3.39) which is statistically significant, indicating that a positive association between estimated change rate of height and risk of T1D diagnosis is found after the covariates of the country of residence, gender, HLA genotype, and FDR status are adjusted in the model. We also find that HR=exp(α_5_) = 1.40 with 95% CI (1.06, 1.86) for HLA genotype and HR=exp(α_6_) = 1.54 with 95% CI (1.08, 2.14) for FDR are significantly associated with a higher risk of T1D. However, gender is not significantly associated with the risk of T1D.

## 4. Simulation Studies

In order to evaluate the performance of the introduced MVJ models and methods, the following limited simulation studies are conducted. The design of the simulated data mimics that of the diabetes data used in Section 3. Specifically, we choose the sample size *n* = 500 and assume that each individual has 32 scheduled longitudinal values. The time points of measurement are mimicked from the real application, and the true values of parameters are selected as follows. $\beta_{1}^{†T}={\left(\beta_{01},\beta_{11},\beta_{21},\beta_{31}\right)}^{T}={\left(59,11,-0.3,0.1\right)}^{T}$, $\beta_{2}^{†T}={\left(\beta_{02},\beta_{12},\beta_{22},\beta_{32}\right)}^{T}={\left(6,2,-0.5,-0.5\right)}^{T}$, $\Upsilon ={\left(\upsilon_{1},\upsilon_{2},\upsilon_{3},\upsilon_{4}\right)}^{T}={\left(-0.2,1,0.4,0.3\right)}^{T}$, and $\alpha ={\left(\alpha_{1},\alpha_{2},\alpha_{3},\alpha_{4},\alpha_{5},\alpha_{6}\right)}^{T}={\left(0.7,0.8,-0.3,-0.4,0.3,0.4\right)}^{T}$. Longitudinal data are simulated based on Equation (6), with each model including individual random intercepts and random slopes with $b_{i}={\left(b_{i01},b_{i11},b_{i02},b_{i12}\right)}^{T}\sim N\left(\text{0},\text{diag}\left(1,1,1,1\right)\right)$, correlation is induced between the two longitudinal outcomes by generating the random intercepts and slopes for each outcome from the multivariate normal distribution; we simulate the model errors ϵ_*ijk*_ under weight sub-model with Γ(2, 0.8) distribution and height sub-model with Γ(1, 0.5) which yield skewed distribution, respectively. To generate the event time data, the constant baseline hazard of 0.1 is set, and an exponential distribution with a mean of 0.1 is used to generate censoring time. The covariates in the survival model are simulated depending on variable types. For example, gender is simulated from a Bernoulli distribution with *p* = 0.5, etc. According to the settings described above, due to the heavy computational burden, we generate 50 data sets, which are fitted by Models N and SN. It is noted that the prior distributions are all close to non-informative similar to those in real data analysis. Thus, the results are expected to be somewhat robust with respect to prior distributions.

Table 2 summarizes the simulation results including the true parameter (TP) values, percent bias (defined by 100 × bias_*l*_/|TP_*l*_|) and percent mean-square-error (MSE) (defined by $100\times {\sqrt{MSE}}_{l}/|{\text{TP}}_{l}|$) of fixed-effects **β**, Υ and **α**. First, it is seen that the estimated parameters in height and weight for our two longitudinal outcomes, which means extending the univariate joint model to MVJ modeling allow us to incorporate more information and improves the efficiency in estimation. Second, for the multivariate linear mixed-effects sub-model, we find that, in comparison to Models SN and N, Model SN generally outperforms Model N in terms of smaller bias and MSE. For all the scenarios considered in this simulation study, it is seen that all estimated biases for β_21_, β_31_, and β_22_ are negative, suggesting that these parameters are underestimated, while estimated biases for β_01_, β_11_, β_12_, and β_02_ are positive, indicating that these parameters are overestimated. We note that the larger bias of the growth rate of height and weight is reasonable, and this is consistent with the results from the real data analysis. The average estimates of skewness parameters δ_1_ = 1.44 for height and δ_2_ = 1.57 for weight in Model SN indicate a departure from (symmetric) normal distribution. Finally, for the estimated parameters in the survival sub-model, Model SN obviously outperforms Model N for the association parameters and baseline covariates except υ_1_, υ_2_, and υ_3_. Some parameters in the survival sub-model are slightly overestimated and some parameters are slightly underestimated in both models. In summary, the simulation results confirm the importance of accounting for the non-normality of the data. This suggests that adopting the assumption of normal distribution may lead to inaccurate inference on fixed-effects of interest, in particular, when longitudinal data exhibit non-normal features.

**Table 2:** Summary of true parameter (TP) values, estimated parameters, Bias, and MSE for Models N and SN based on 50 simulated data sets.

**Model**		**Model SN**			**Model N**		
**Parameter**	**TP**	**EST**	**Bias**	**MSE**	**EST**	**Bias**	**MSE**
**Parameter estimates in longitudinal sub-model**							
β_01_	59	59.61	1.04	0.64	59.61	1.04	0.64
β_11_	11	11.81	7.37	6.21	11.85	7.74	0.60
β_21_	–0.3	–0.30	–1.56	–0.01	–0.30	–1.60	0.01
β_31_	0.1	–0.10	–1.32	0.01	0.09	-1.16	0.01
β_02_	6	6.81	13.52	11.21	6.80	13.41	10.84
β_12_	2	2.66	33.08	32.11	2.80	39.96	32.24
β_22_	–0.5	–0.46	8.42	–1.02	–0.45	9.99	1.13
β_32_	–0.5	–0.54	–9.60	0.73	–0.55	–10.67	0.72
δ_1_		1.44	–	–	–	–	–
δ_2_		1.57	–	–	–	–	–
**Parameter estimates in survival sub-model**							
υ_1_	–0.2	–0.19	2.80	0.10	–0.20	0.11	0.08
υ_2_	1	0.66	–33.60	11.54	0.67	–33.22	11.31
υ_3_	0.4	0.29	–27.42	4.50	0.31	–23.54	3.52
υ_4_	0.3	0.24	–19.74	1.63	0.24	–19.80	1.64
α_1_	0.7	0.74	6.04	0.39	0.75	6.55	0.35
α_2_	0.8	0.90	12.36	1.70	0.91	14.10	1.66
α_3_	–0.3	–0.15	49.99	10.08	–0.14	52.77	8.44
α_4_	–0.4	–0.20	49.99	10.08	-0.19	50.63	10.27
α_5_	0.3	0.30	–0.88	0.37	0.24	–1.94	0.06
α_6_	0.4	0.32	–19.28	1.54	0.32	–19.78	1.58

*EST is the average of estimates, Bias and MSE are quantified by the percent bias = 100 × bias_l_/|TP_l_| and percent $\sqrt{\text{MSE}}=\text{100}\times {\sqrt{\text{MSE}}}_{l}/|{\text{TP}}_{l}|$, respectively*.

## 5. Conclusion

In this study, we propose a Bayesian MVJ model with multiple longitudinal responses and survival processes. The MLME sub-model and the survival sub-model are linked through the random-effects which are served to characterize the underlying subject-specific longitudinal process (Xu, 2021). We also consider some important data features such as non-normality which may impact the discovery of the true disease diagnosis progression. Comparing with the classic frequentist's methods, a Bayesian approach is powerful when the dimension of parameters is high in the complicated MVJ modeling. Although the joint modeling for longitudinal and time-to-event data has been an active area of statistical methodological study (Huang et al., 2011; Chen et al., 2014; Huang and Chen, 2016; Zhang and Huang, 2020), this paper extends to investigate joint models for survival and multivariate longitudinal data with SN distribution, accounting for multiple data features, simultaneously. Although this article is motivated by a diabetes study, the innovations of the proposed multivariate joint models and methods may help applied researchers analyze complicated longitudinal and survival data under a wide range of applications.

The proposed MVJ model offers some advantages in comparison with traditional joint models. First, the majority of joint models focus mainly on a single longitudinal outcome associated with the survival endpoint. However, in many clinical and observational studies, multiple longitude data are collected together and they may be significantly correlated. The MVJ model proposed here is able to reduce the bias and can increase the efficiency in parameter estimation. These interesting findings have important clinical indications. Our results suggest that there is a positive association between rates of growth and risk of T1D (i.e., there is an increased risk of T1D for larger height and weight at baseline). Second, due to its importance to measure height and weight appropriately when they show non-normality with heavy tails, this article considers two statistical models (Models N and SN) with different scenarios. We find that Model SN is favorable to model N. In model SN, the estimates of skewness parameters δ_1_ and δ_2_ are statistically significant for height and weight, indicating that the skewness with a heavy tail exists in height and weight measurements. Therefore, the MVJ model with the SN distribution provides more efficient and accurate estimates of parameters, and, thus, serves as a better alternative to the normal distribution-based model which is widely adopted.

We apply the Bayesian MVJ modeling approach to analyze the diabetes data set in this article. The results demonstrate the use of the MVJ model to investigate how the patterns of longitudinal height and weight trajectories are associated with the risk of T1D. Furthermore, our results indicate that it is of importance to consider the MVJ model with the skewed distribution in order to obtain more accurate and less biased parameter estimates in the presence of non-normal features in longitudinal height and weight data. Although this article is motivated by a diabetes study, the fundamental concepts of the proposed Bayesian modeling method should have generally broader applications in practice whenever the two different sources of dependence among longitudinal data over time and between longitudinal and survival variables are presented, and the relevant technical specifications are met (Chen et al., 2021). Our models may have the potential to extend to more complicated models. For example, (i) the missing data mechanism may be considered by introducing a non-ignorable missing data model for longitudinal measurements (Huang, 2016; Huang and Chen, 2016). (ii) although a single-type event time is investigated only in this article, the developed MVJ model may be extended to accommodate competing risks survival data in the presence of multiple “failure” types of events (Elashoff et al., 2008; Hu et al., 2009). Although these interesting topics are beyond the focus of this paper, they are warranted in our research pipeline under investigation.

## Data Availability Statement

The data are available upon request to the authors.

## Author Contributions

YH: research concept and design. YH, JC, and LX: collection and/or assembly of data and statistical analysis. YH, JC, LX, and N-ST: data analysis and interpretation, writing the article, critical revision of the article, and final approval of article. All authors contributed to the article and approved the submitted version.

## Funding

This study was partially supported by the High-End Foreign Experts Program of Yunana Province Program to YH, the National Natural Science Foundation of China grant (81671633) to JC, and the National Natural Science Foundation of China grant (11731011) to N-ST.

## Conflict of Interest

The authors declare that the research was conducted in the absence of any commercial or financial relationships that could be construed as a potential conflict of interest.

## Publisher's Note

All claims expressed in this article are solely those of the authors and do not necessarily represent those of their affiliated organizations, or those of the publisher, the editors and the reviewers. Any product that may be evaluated in this article, or claim that may be made by its manufacturer, is not guaranteed or endorsed by the publisher.
