# Supplementary material for: Bayesian Joint Modeling of Multivariate Longitudinal and Survival Data With an Application to Diabetes Study
Source: Front Big Data. 2022 Apr 27;5:812725. doi: 10.3389/fdata.2022.812725 (PMC9094046; doi:10.3389/fdata.2022.812725)
Supplement: Supplementary file 1 [file Presentation_1.pdf]

APPENDIX. WINBUGS PROGRAM CODES FOR MODEL SN

```
########################################################################
#### R program codes
########################################################################
 library(arm)
 library(R2WinBUGS)
 data<-list(......)
 inits<-list()  # Run three chains
 inits[[1]]<-list(......)
 inits[[2]]<-list(......)
 inits[[3]]<-list(......)
 parameters<-c(......)
 hw.t1d.BVJM <-bugs(data, inits, parameters,"hw.t1d.BVJM_SN.txt",n.chains=3,
            n.thin=20, n.iter=30000,n.burnin=10000,bugs.seed=654321,
            bugs.directory="C:\\Users\\WinBUGS14",DIC=TRUE,debug=TRUE)
########################################################################
#### Start WinBUGS codes: hw.t1d.BVJM_SN.txt
########################################################################
model {
for (i in 1:n)   # n total number of subjects
{
# random effects for bivariate model with normal distribution
  for (k in 1:4)
     { b1[i,k] <- 0}
      b[i,1:4] ~ dmnorm(b1[i,1:4],Omega[,])

## individual parameters
 beta11[i] <- beta[1]+b[i,1]
 beta21[i] <- beta[2]+b[i,2]
 beta31[i] <- beta[3]+b[i,3]
 beta41[i] <- beta[4]+b[i,4]

} #End of i

## (1) Event time models(Cox regression model)###
for(i in 1:n)
  # set up data
        {
  for(j in 1:Tnum)    #Tnum=42
              {
       # risk set = 1 if obs.t >= t
 risk[i,j] <- step(obs.t[i] - t[j] + eps)
# counting process jump = 1 if obs.t in [ t[j], t[j+1] )
#      i.e. if t[j] <= obs.t < t[j+1]
 dN[i, j] <- risk[i, j] * step(t[j + 1] - obs.t[i] - eps)*fail[i]
      }
}
  # Cox regression model
for(j in 1:Tnum)
        {
   for(i in 1:n)
             {
       dN[i, j] ~ dpois(Idt[i, j])                        # Likelihood
```

```
       Idt[i, j] <- risk[i, j] *
   exp(theta[1]*beta11[i]+theta[2]*beta21[i]+
       theta[3]*beta31[i]+theta[4]*beta41[i]+
   alpha[1]*T[i,5]+alpha[2]*T[i,6]+alpha[3]*T[i,7]+
   alpha[4]*T[i,8]+alpha[5]*T[i,9]+alpha[6]*T[i,10])*dL0[j] #Intensity
             }

   dL0[j] ~ dgamma(mu0[j], c0)
   mu0[j] <- dL0.star[j]*c0               # prior mean hazard
}
         #####coefficients
           c0 <- 0.1
   r <- 5
   for (j in 1 : Tnum) { dL0.star[j] <- r * (t[j + 1] - t[j]) }

for(j in 1:N)   # N=total number of longitudinal measurements
{

## (2) Modelling bivariate longitudinal model with SN distribution
  wh[j,1]<-beta11[y[j,1]]+beta21[y[j,1]]*y[j,3]
           +gamma1[1]*y[j,3]*y[j,3]+gamma2[1]*y[j,6]
  wh[j,2]<-beta31[y[j,1]]+beta41[y[j,1]]*y[j,3]
           +gamma1[2]*y[j,3]*y[j,3]+gamma2[2]*y[j,6]
    #y[j,1]=id, y[j,3]=age, y[j,6]=female

##SN-distribution
w[j] ~ dnorm(0, 1) I(0,)             ##SN
mean.wh[j,1]<-wh[j,1]+delta1*(w[j]-0.798)
mean.wh[j,2]<-wh[j,2]+delta2*(w[j]-0.798)

y[j,4:5]~dmnorm(mean.wh[j,1:2], Omega1[,])
Y.pred[j,1:2]~dmnorm(mean.wh[j,1:2], Omega1[,])

#Fitted values and residuals
 fit.h[j]    <- mean.wh[j,1]
 resid.h[j]  <- y[j,4]-fit.h[j]
    ssr.h[j] <- pow(resid.h[j],2)   # squares of residuals of height

 fit.w[j]    <- mean.wh[j,2]
 resid.w[j]  <- y[j,5]-fit.w[j]
# sresid.w[j] <- sqrt(Omega1[2,2])*resid.w[j]
    ssr.w[j] <- pow(resid.w[j],2)   # squares of residuals of weight

}#end of N


# Prior distributions of the hyperparameters
#(1) Coefficients
for(k in 1:4){theta[k] ~dnorm(0,0.01)}
for(k in 1:4){beta[k]  ~dnorm(0,0.01)}
for(k in 1:6){alpha[k] ~dnorm(0,0.01)}
for(k in 1:2){gamma1[k]~dnorm(0,0.01)}
for(k in 1:2){gamma2[k]~dnorm(0,0.01)}
```

```
#(2). Variance-covariance matrice for model error
 Omega1[1:2,1:2] ~dwish(R1[,],3)
 v1[1:2,1:2] <- inverse(Omega1[,])

#(3) Variance-covariance matrix for random-effects vector
 Omega[1:4,1:4]~dwish(R[,],5)
 v[1:4,1:4]<-inverse(Omega[,])

#(4) Skewness parameters
 delta1~dnorm(0,0.01)
 delta2~dnorm(0,0.01)
}
## End of model
```
